# Exploring the link between physician burnout and intentions to retire early

**DOI:** 10.1186/s12889-025-24841-3

**Published:** 2025-10-21

**Authors:** Franziska Ulrike Jung, Erik Bodendieck, Alexander Pabst, Melanie Luppa, Steffi G. Riedel-Heller

**Affiliations:** 1 Institute of Social Medicine, Occupational Health and Public Health (ISAP), Faculty of Medicine, Leipzig University, Ph.-Rosenthal-Str. 55, Leipzig, 04103 Germany; 2 General Practice, Dresdner Straße 34a, Wurzen, 04808 Germany

**Keywords:** Physicians, Burnout, Retirement, Workload, Longitudinal studies

## Abstract

**Background:**

In the light of physician shortage and increasing demands of medical services, losing physicians due to early retirement is becoming a significant problem for healthcare systems worldwide. The aim of this study was to investigate retirement plans and the relationship between burnout and retirement planning.

**Method:**

For the purpose of the current study, a sample of *n* = 320 physicians working in patient care was analyzed in 2020 (Baseline) and repeatedly in 2024 (Follow-up). The questionnaire included the Copenhagen Burnout Inventory, measuring overall burnout and sub dimensions (personal, patient- and work-related). One single item assessed retirement planning (earlier vs. later/regular). Mixed-effects generalized linear models with binomial family and logit-link were applied, adjusting for sociodemographic characteristics.

**Results:**

In terms of retirement planning, 42% of physicians in 2020 and 39% pf physicians in 2024 were planning to retire early. The highest prevalence of burnout was found with regard to personal burnout at both time points. In addition, a significant association between time retirement planning and burnout was found: for a one-unit increase in overall burnout, a 12% increase in the odds of desiring early retirement may be expected when controlling for sociodemographics (*p* = 0.001). Specifically, an increase in work-related burnout was significantly related to a 7.1% higher odds of early retirement intention (p = 0.005) while no relationship was found for personal or patient-related burnout.

**Conclusion:**

Current analyses indicate that burnout constitutes a significant risk factor for the early retirement of physicians and warrants appropriate attention in workforce planning. In order to avoid negative consequences for the broader healthcare system, it is important to establish interventions that help physicians to cope with high workload. Future studies are needed to better understand decisions regarding retirement among physicians.

**Supplementary Information:**

The online version contains supplementary material available at 10.1186/s12889-025-24841-3.

## Introduction

### Healthcare and physician shortage

These days, the healthcare sector worldwide is facing several challenges. The demand for health care is steadily increasing, driven by a rise in chronic conditions such as obesity and diabetes, as well as demographic changes, with a significant proportion of the population expected to be over 65 years old in the coming years [1, 2]. At the same time, a significant number of physicians in Germany and beyond Europe have already reached the age of 50 and may be approaching retirement [3, 4]. Even if the number of new physicians increases every year, it cannot replace the number of physicians who are leaving the workforce to this extent [5, 6]. For comprehensive health care planning, it is important to investigate physicians’ well-being and health (or ability to work) and analyse which factors may influence physicians’ intention to retire (in the long term). Even though the legal retirement age may vary across countries (currently between 65 and 67 years of age in Germany), the issue is of great relevance nationwide.

Although, there is no clear definition of burnout so far, it is characterized by feelings of (physical or psychological) fatigue and exhaustion as a result of emotional demanding situations across various domains at work [7–10]. According to Kristensen et al. [9] a multifaceted evaluation of burnout may be helpful to identify factors that contribute to its development and progression. In this context, the following three distinct dimensions of burnout have been proposed in order to evaluate the complex association between fatigue and work [9]:

1. “the degree of physical and psychological fatigue and exhaustion experienced by the person” (personal burnout)
2. “the degree of physical and psychological fatigue and exhaustion that is perceived by the person as related to his or her work” (work-related burnout) and
3. “The degree of physical and psychological fatigue and exhaustion that is perceived by the person as related to his or her work with client” (client- or patient-related burnout).

Previous studies suggest that doctors are often overworked and have an increased risk of burnout. The reasons for this are manifold: confrontation with death and suffering, sleep deprivation, time pressure, irregular or excessively long working hours, less autonomy and excessively high patient expectations have been linked to burnout [11–14]. So far, studies related to burnout and mental health of physicians in Germany are scarce compared to other countries [15]. However, it has been suggested by studies with small sample sizes, that German physicians working in palliative care, as well as GPs working in single practices (compared to group practices) are more likely to suffer from burnout symptoms [16, 17]. Previous research has shown that burnout may be linked to negative outcomes for both physicians’ well-being and patient safety [18–23], and it may also influence retirement planning, particularly regarding the timing of retirement [24].

According to the literature, the relationship between burnout and retirement may depend on several sociodemographic characteristics, such as age, gender, marital status and parenthood. Older physicians have been shown to exhibit less burnout compared to their younger colleagues [25], however, it is unknown whether age-related retirement decisions as a result of burnout exist. Furthermore, female physicians may be more likely to exhibit greater burnout risk, and in addition, retire earlier than their male counterparts [24, 25]. Married individuals often have more emotional and instrumental support, which can protect against burnout [26]. On the other hand, decisions about retirement are often made in coordination with a spouse’s career and health status [27], potentially confounding the association with burnout. Parenthood may be associated with work-family conflicts, increasing the risk for physician burnout [28]. At the same time, having children—particularly younger or dependent ones—can delay retirement due to financial and caregiving responsibilities or to compensate for financial losses due to past childcare periods. Therefore, taking into account sociodemographic differences is important.

Previous studies have found that physician burnout leads to significant economic costs in both Canada and the United States. In Canada, the estimated annual cost is approximately 213 million Canadian dollars, primarily due to early retirement and reduced working hours. In the United States, a conservative model estimates annual costs of about 4.6 billion US dollars, mainly driven by physician turnover and decreased clinical productivity [29, 30]. In the light of physician shortage across mainly medical domains and in addition to the increasing numbers of physicians that will reach usual retirement age within the next years, it is important to explore factors that may be associated with the decision to retire. Building on prior research, this study focused on the relationship between burnout and the intention to retire. In line with Kristensen et al. [9], who emphasize the importance of distinguishing between specific sub-dimensions of burnout—namely personal, work-related, and client-/patient-related exhaustion—we aimed to examine burnout in a more nuanced and differentiated manner. In this context, we investigated whether the associations with retirement planning differ depending on the type of exhaustion experienced, i.e., personal versus work-related or client-/patient-related. To our knowledge, this is the first study to explore the associations between general burnout and its distinct dimensions with retirement planning among physicians, using longitudinal data collected at baseline (2020) and follow-up (2024). Furthermore, we accounted for the potential influence of demographic and contextual factors, including age, gender, and medical work setting (e.g., hospital versus other healthcare environments), on burnout and retirement intentions.

## Method

### Recruitment and study population

**Fig. 1:**
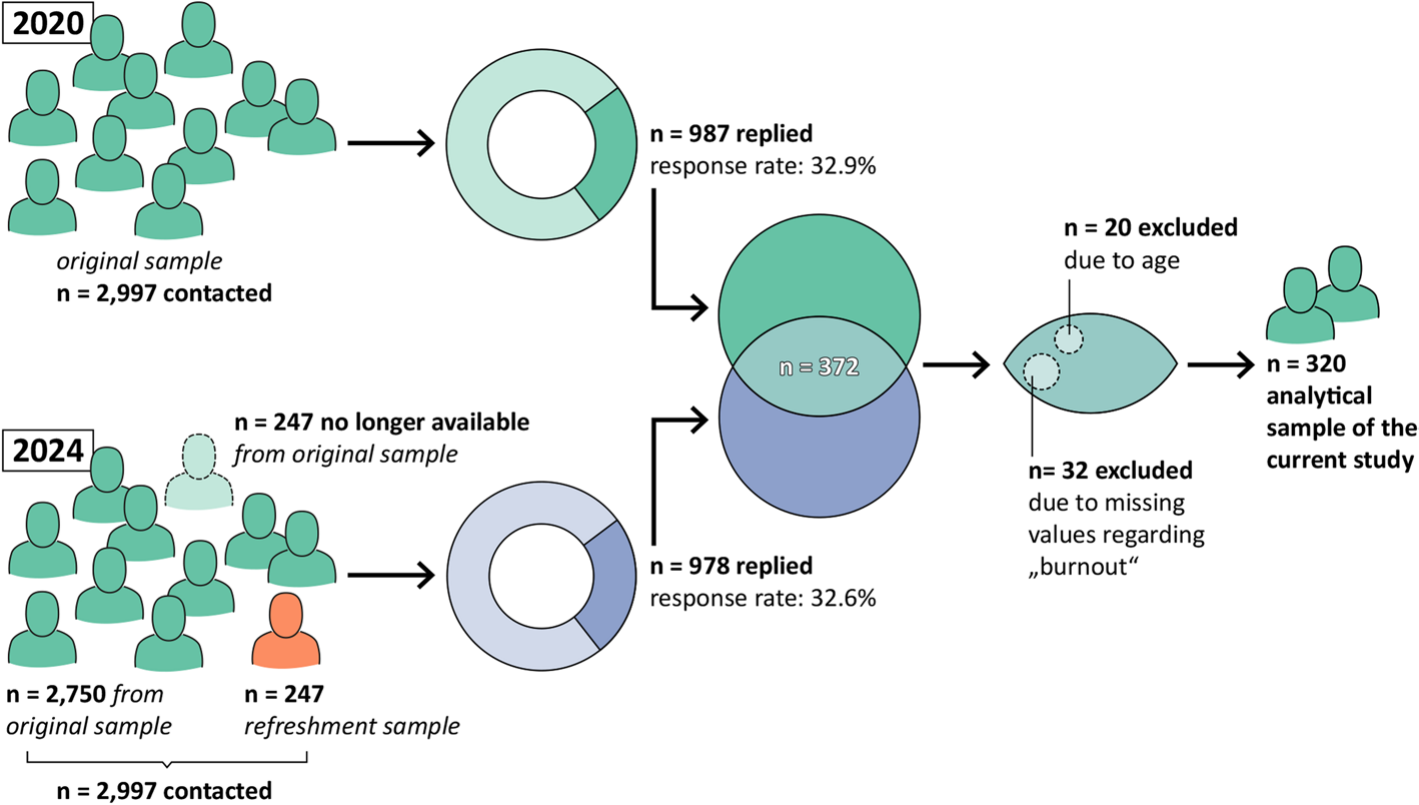
Sampling and study procedure

The data sample originates from a survey focusing on working hours and retirement among physicians. The sampling process followed methodological principles, including the formation of nine distinct age cohorts: physicians aged 26–30, physicians aged 31–35, and so forth. According to this recruitment plan, the target was set at 100 respondents per cohort to ensure adequate statistical power and to secure a sufficient number of participants for subsequent follow-up in 2024. Assuming an average response rate of approximately 30%, about 333 physicians were randomly selected and contacted in each age group, yielding a total sample of 2,997 across all age cohorts. The questionnaire consisted of validated instruments and own items (paper–pencil, anonymous). In 2020 (Baseline), 32.9% of *n* = 2.997 physicians being contacted by mail replied, resulting in *n* = 987 cases. In 2024, 247 of 2,997 physicians could no longer be contacted, so an additional sample of *n* = 247 (refreshment sample) has been added in order to achieve a sample size of 2,997. Overall, the response rate in 2024 was 32.6%, resulting in *n* = 978 cases at follow-up (Fig. 1).

In 2020 and 2024, participants were asked to generate their own unique identification code (ID) as part of the questionnaire. The ID met strict guidelines, and enabled matching between baseline and follow-up. The ID code included a sequence of letters and numbers based (self-generated identification code): mother’s first name (first and last letter), fathers’s first name (first and last letter), respondent’s first name (first and last letter) and mother’s date of birth (day). This code and similar variants have been used on other studies before [31–33].

However, only physicians who took part in both the baseline survey conducted in 2020 and the follow-up survey in 2024 were included in the current study sample for further analysis (*n* = 372, Fig. 1). Furthermore, cases were excluded from analysis, if the age of the participating physicians at baseline was beyond regular retirement age of physicians in Germany (i.e. 67 years and older, *n* = 20) and due to missing values regarding „burnout “(*n* = 32). Therefore, the final sample included *n* = 320 physicians. Overall sample and sub-sample differed in terms of gender and age (*p* < 0.001 for both), as the sub-sample contained a higher percentage of physicians being female and physicians were younger.

The ethical committee of the Medical Faculty (Leipzig University) approved the study before conduction (ref. number: 478/19-ek).

### Instruments

Apart from socio-demographic characteristics, such as age, gender, marital status and parenthood (yes/no), the questionnaire also included an item on whether they work in inpatient or outpatient care (yes/no).

In order to record their desired time of retirement, the study participants were asked about their timing of retirement (answer options: 1 = *early retirement*, 2 = at *regular age*, 3 = *after the regular retirement age* or 4 = *don't know*) using the following question: “If you could decide freely: When would you choose to retire?” [34]. This item has been used in previous studies investigating intention to retire [35–37].

Burnout was assessed using the Copenhagen Burnout Inventory (CBI) [9]. The scale surveys the perception of the work situation (and the associated stress) using 19 items and a 5-point response scale, adapted for professionals working in the healthcare sector [38]. According to the literature, all items were transformed, ranging from 0 (minimum score, e.g. answer categories “very little/never”) to 100 points (maximum score, e.g. answer categories “very strong/always”)[39, 40]. An average was calculated based on the sum scores. A higher score indicates greater burnout [9]. In line with other studies [40–42], CBI scores may be categorized into no burnout (< 25), low burnout (≥ 25 & < 50), moderate burnout (≥ 50 & < 75) and high burnout (≥ 75 & < 100). The CBI consists of three subscales. Personal burnout included six items (example: “How often do you feel tired?”; from *all the time/always* to *never/almost never*). Work-related burnout consisted of seven items (example: “Does your work put a strain on you?” emotional?”; from *very strong* to *very little/not at all*). Patient-related burnout included six items (example: “Does working with patients put a strain on you?”; from *very strong* to *very little/not at all*) [9, 40]. The items can be found in the appendix. The Cronbach’ alphas of the overall burnout scale (baseline and follow-up) as well as the three sub-scales (baseline and follow-up) ranged from 0.86 to 0.94, showing good and excellent internal consistency.

### Data analysis

Data was analyzed descriptively in order to compare sociodemographic and work-related characteristics as well as the main outcome between baseline and follow-up. With regard to multivariate analyses, the outcome variable (retirement planning) was dichotomized as follows in order to measure likelihood of early retirement: 1 = early retirement and 0 = regular or later retirement. The response category 'I don't know' was not included in this dichotomization. Multilevel mixed-effects generalized linear models with a binomial distribution and logit link function were carried out to determine the associations between retirement planning and burnout, using a random intercept to measure heterogeneity over time and the Huber/White sandwich estimator to adjust standard errors for possible model misspecification [43]. First, a model was run using overall burnout as continuous predictor, adjusting for time (baseline vs. follow-up). Second, this model was extended by additionally taking into account specified control variables (age, gender, marital status, parenthood, working setting, all at both baseline and follow-up). Subsequently, the same models were run for burnout subscales by replacing overall burnout with all three subscales simultaneously as predictors. We report odds ratios, 95%-Confident Intervals (95%-*CI*), as well as *p*-values. A *p*-value threshold of less than or equal to 0.05, was used to determine statistically significance. Stata SE Version 16 provided the basis for all calculations [44].

## Results

Information on sociodemographic and work-related characteristics can be found in Table 1. The majority of the sample was female (65.3%) and the mean age at baseline was 43.7 years. The sample significantly differed between baseline and follow-up regarding parenthood (having children: 75.6% in 2020 vs. 86.6% in 2024, *p* < 0.001) and their medical setting (working in a hospital: 60.3% in 2020 vs. 52.2% in 2024, *p* < 0.001). No statistical significant differences could be observed in terms of overall burnout or the three burnout sub-scales (between baseline and follow-up, Table 1). However, the proportion of physicians planning to retire early slightly decreased from 42% in 2020 to 39% in 2024.

**Table 1:** Baseline—Characteristics of the study sample (*n* = 320)

	**Baseline**	**Follow-up**	**Significance**
**Sociodemographic Factors**			
Age (in years), M(SD)	43.7 (SD: 10.8, range: 25–66)	47.6 (SD: 10.7, range: 29–70)	*p* < 0.001
Sex, n(%)			/
Female	209 (65.3%)		
Having children, n(%)			
Yes	242 (75.6%)	277 (86.6%)	*p* < 0.001
No	77 (24.1%)	42 (13.1%)	
Missing information	1 (0.3%)	1 (0.3%)	
Marital status, n(%)			
Single	27 (8.4%)	35 (11.0%)	*p* = 0.052
Widowed	2 (0.6%)	4 (1.3%)	
Separated/not living together	17 (5.3%)	19 (6.0%)	
Married/in a relationship	274 (85.6%)	261 (81.82%)	
**Work-related factors**			
Working in a hospital, n(%)			
Yes	194 (60.3%)	167 (52.2%)	*p* < 0.001
No	125 (39.1%)	142 (44.4%)	
Missing information	1 (0.3%)	11 (3.4%)	
**Burnout**			
Burnout, M(SD)^a^			
Overall	33.8 (SD 15.3)	34.5 (SD: 16.6)	*p* = 0.363
No burnout (< 25)	96 (30.0%)	97 (30.3%)	*p* = 0.320
Low burnout (≥ 25 & < 50)	175 (54.7%)	162 (50.6%)	
Moderate burnout (≥ 50 & < 75)	45 (14.1%)	56 (17.5%)	
High burnout (≥ 75 & < 100)	4 (1.3%)	5 (1.6%)	
Personal	44.4 (SD: 17.4)	45.7 (SD: 19.2)	*p* = 0.144
Work-related	33.9 (SD: 16.7)	34.4 (SD: 18.4)	*P* = 0.874
Patient-related	22.9 (SD: 18.9)	23.5 (SD: 18.6)	*P* = 0.289
**Retirement**			
Intention to retire early, n(%)	135 (42.2%)	125 (39.1%)	*P* = 0.419
Intention to retire regularly, n(%)	84 (26.3%)	81 (25.3%)	
Intention to retire later, n(%)	41 (12.8%)	51 (15.9%)	
Don’t know, n(%)	59 (18.4%)	60 (18.8%)	
Missing information, n(%)	1 (0.3%)	3 (0.9%)	

*M* Mean, *SD* Standard deviation

^a^burnout scores may range from 0 to 100 with higher scores indicating greater burnout

### Regression analyses

Table 2 summarized the regression model of overall burnout and likelihood of early retirement. According to the results, plans for early retirement increases by 12.4% with every one-unit increase in overall burnout (odds ratio = 1.124, *p* < 0.001), independent of time. Moreover, retirement planning does not change over time, and remained stable between 2020 and 2024 (odds ratio = 0.668, *p* = 0.352).

**Table 2:** Multilevel mixed-effects generalized linear model for the association between symptoms of overall burnout and likelihood of early retirement (*n* = 297)

**Retirement planning**				
	Odds Ratio	*p*-value	95% CI	
Burnout	**1.124**	**< 0.001**	**1.062**	**1.190**
Time (ref. Baseline)	0.668	0.219	0.352	1.266

*CI* confidence interval

As can be seen in Table 3, the effect of overall burnout on retirement planning remained significant after adding covariates to the model. The results show, that likelihood to retire early increases by 11.9% with every one-unit increase in overall burnout (odds ratio = 1.119, *p* < 0.001). Again, no significant change over time regarding retirement planning was observed (odds ratio = 0.629, *p* = 0.170).

**Table 3:** Multilevel mixed-effects generalized linear model for the association between symptoms of overall burnout and retirement planning (with covariates, *n* = 296)

**Retirement Planning**				
	Odds Ratio	*p*-value	95% CI	
Burnout	**1.119**	**< 0.001**	**1.060**	**1.180**
Time (ref.: Baseline)	0.629	0.170	0.324	1.220
Age	0.986	0.548	0.941	1.033
Sex (ref. female)	0.559	0.279	0.195	1.601
Having children (ref. yes)	0.413	0.161	0.120	1.422
Marital Status (ref. not married)	0.903	0.874	0.258	3.161
Working in a hospital (ref. yes)	0.459	0.119	0.172	1.221
	Wald chi^2^ = 17.54; *p* = 0.014			

* CI* Confidence Interval

In addition, each of the three dimensions of burnout (work-related, patient-related and personal) were separately tested. The results can be found in Table 4.

**Table 4:** Multilevel mixed-effects generalized linear model for the association between all three dimensions of burnout and retirement planning (univariate,* n* = 297)

**Retirement Planning**				
	Odds Ratio	*p*-value	95% CI	
Personal burnout	**1.042**	**0.042**	**1.001**	**1.085**
Work-related burnout	**1.073**	**0.005**	**1.021**	**1.127**
Patient-related burnout	1.004	0.817	0.972	1.036
Time (ref.: Baseline)	0.661	0.198	0.352	1.242
	Wald chi^2^ = 19.93; *p* < 0.001			

*CI* Confidence Interval

With regard to the three dimensions of burnout, it was found that personal and work-related, but not patient-related burnout was significantly associated with retirement planning. In other words, with every one-unit increase in personal burnout, intention to retire early increased by 4.2% (odds ration = 1.042, *p* = 0.042). Similar, with every one-unit increase in work-related burnout, the likelihood of early retirement increased by 7.3% increase (odds ratio = 1.073, *p* = 0.005, Table 4).

The results of the multivariate regression analysis are shown in Table 5. After adjusting for sociodemographic and work-related variables, similar results were observed. In this context, likelihood to retire early increases by 7.1% with every one-unit increase in work-related burnout (odds ratio = 1.071 *p* = 0.005). However, no significant effect was found in relation to personal (odds ratio = 1.035, *p* = 0.093) or patient-related burnout (odds ratio = 1.007, *p* = 0.681).

**Table 5:** Multilevel mixed-effects generalized linear model for the association between burnout and retirement planning (with covariates, *n* = 296)

**Retirement Planning**				
	Odds ratio	*p*-value	95% CI	
Personal burnout	1.035	0.093	0.994	1.077
Work-related burnout	**1.071**	**0.005**	**1.021**	**1.125**
Patient-related burnout	1.007	0.681	0.974	1.041
Time (ref.: Baseline)	0.634	0.172	0.330	1.219
Age	0.987	0.606	0.941	1.036
Sex (ref. female)	0.659	0.445	0.226	1.924
Having children (ref. yes)	0.469	0.231	0.136	1.618
Marital Status (ref. not married)	0.891	0.856	0.255	3.105
Working in a hospital (ref. yes)	0.540	0.222	0.201	1.450
	Wald chi^2^ = 21.37; *p *= 0.011			

*CI* Confidence Interval

## Discussion

The aim of this study was to investigate the association between burnout and retirement planning in a broad sample of repeatedly assessed physicians. Our findings show that a substantial proportion of physicians planned to retire early—42% in 2020 and 39% in 2024—indicating that early retirement remains a relevant issue in the healthcare sector with implications for workforce sustainability and patient care. While the overall burnout scores measured by the CBI remained relatively stable over time (33.8 in 2020 and 34.5 in 2024), physicians with intentions of early retirement reported significantly higher levels of personal and work-related burnout. In contrast, patient-related burnout did not show a significant association with retirement intentions. These results suggest that specific dimensions of burnout—particularly personal and work-related exhaustion—may play a role in physicians’ decisions to retire early [24]. Recent studies have shown that the percentage of physicians who plan to retire early range between 8 and 48%, depending on medical specialty, age, work-related factors, psychological factors and job satisfaction [11, 24, 45–48]. The overall burnout score of the CBI was 33.8 in 2020 and 34.5 in 2024, similar to other studies reporting CBI scores among physicians [49, 50]. The prevalence of personal burnout was slightly higher compared to similar studies [51, 52]. In this context, it has been shown before, that health care personal is more likely to be affected by personal burnout, compared to work- or patient-related burnout [40, 52, 53]. However, compared to nurses, physicians may be more affected by work-related burnout, going align without findings [52]. High risk of burnout among physicians is of great relevance, as it has been shown to be linked to lower well-being, but also negative consequences in terms of patient safety and quality of care [11, 54]. However, results of the current study show no significant change in overall burnout, as well as regarding the sub-dimensions personal, patient-related as well as work-related burnout between 2020 and 2024. Previously, it has been argued that burnout is a rather chronic condition, characterized by stable components [12]. On the other hand, it has been suggested, that personal factors (i.e. coping mechanisms) as well as job demands (i.e. patient demands, job resources) may shape within-person developmental processes with regard to burnout [12, 55, 56]. Hence, individual differences in the development and dynamics of burnout symptoms warrant further investigation in future studies.

With regard to the mixed-effects binomial models, the current study found that there is a significant association between burnout and retirement intentions. In fact, greater symptoms of overall burnout were associated with early retirement intention (compared to regular or late retirement). The fact that physician burnout may be associated with greater odds for retirement has been shown in a cross-sectional study before [57], as well as during the COVID-19 pandemic [58]. In addition, our study manifested these findings longitudinally, and showed that personal burnout and work-related burnout were specifically associated with higher odds of early retirement, but not patient-related burnout. However, after adding covariates to the model (such as age, gender or marital status), these findings remained significant with regard to work-related burnout. The association between burnout and (early) retirement may be explained by consequences linked to burnout in physicians. As work-related burnout increases, it may increase the desire to retire early to escape the stressful work environment. According to a recent systematic review and meta-analysis, chronic workplace stress and burnout can lead to feelings of being overwhelmed or detached from work, resulting in job dissatisfaction and turnover intention or regretting career choices [54]. It may be possible that physicians will not only regret career choice or leave their job, but also want to leave patient care as soon as possible by retiring early. A study by Schaufeli and Bakker (2004) shows that burnout is associated with a variety of health problems that can impair work ability and promote turnover intention. Interestingly, in our study, work-related burnout (i.e. physical and psychological exhaustion/fatigue related to work) was significantly associated with the intention of early retirement. Previously, work-related aspects, such as working hours (i.e. shift work, after-hours charting and time pressure) have been linked to increased risk for burnout and distress in physicians, lower job satisfaction and higher intention to leave [59–62]. In addition, work-related factors may be more relevant in elevating burnout risk, compared to patient-related aspects or the interaction between physicians and patients [63]. Therefore, the concept of burnout is rather complex, even though we found evidence for the strong link between burnout and retirement planning – independent on sociodemographic or work-related characteristics, such as outpatient or inpatient settings. Previously, it has been suggested that the relationship between burnout and retirement planning may depend on several sociodemographic characteristics, such as age, gender, marital status and parenthood [24–26]. However, multivariate analyses in the current study did not reveal any significant associations regarding these aspects. As already mentioned, the initially significant association between personal burnout and early retirement intentions was no longer observed after adjusting for sociodemographic covariates such as age, gender, marital status, and having children. This suggests that these factors may confound or mediate the relationship between personal burnout and retirement planning. For example, individuals may experience different levels of burnout and intentions to retire early, depending on personal risk and protective factors associated with burnout and early retirement [21, 24, 64]. Clearly, more research is needed to clarify, whether and how individual differences—such as sociodemographic characteristics—may contribute to the association between burnout and intention to retire among physicians.

It is still unclear whether the intention of physicians to leave the profession or retire early will translate into action. With regard to other professions, it has been shown that being dissatisfied with work (mainly due to adverse working conditions) was not only related with the intention to retire, but also led to actual retirement [65]. Therefore, prospective studies are needed in order to monitor not only changes or developments of intention to retire, but also the actual timing of retirement, pre-retirement conditions and reasons for timing. In addition, the impact of financial and time-related aspects, such as socioeconomic status, income or working hours/working time arrangements, may be addressed by future studies in order to better understand the process of retirement planning. Burnout-related turnover and early retirement has been shown to be costly in terms of health care expenditures [66]. Retaining doctors – especially nearing the time of retirement is important, because otherwise their experience-based knowledge cannot be passed onto the next generation and because they could be helpful in filling a gap in times of physician shortage. There is evidence, that physicians would be willing to work beyond regular retirement age, if working hours and on-call (for hospital doctors) as well as job intensity (for GPs) are adjusted according to their needs [67].

### Strength and weaknesses

The current study does have its drawbacks. Physicians with specifically high workload or work-related stress may not have participated in this study, due to a lack of time. Therefore, burnout rates may be higher. We based our analysis on a sub-sample, as only a portion of physicians participated at both time points (2020 and 2024). Consequently, the results may be subject to bias, given that the sub-sample differed significantly from the overall sample in terms of age and gender. Due to anonymization and privacy restrictions, we were not able to compare responders and non-responders (i.e. those who did not participate in either 2020 or 2024). In addition, due to the small sample size, it was not possible to differentiate between different medical fields. Previously, it has been shown, that surgeons exhibit some of the highest rates of workload and burnout compared to other fields [68]. As a result, it is not possible to draw conclusions about individual differences that may be associated with physicians' medical backgrounds. Our study did not include certain potentially influential factors, such as full-time versus part-time status, actual hours worked per week, the individual’s financial situation (e.g., economic feasibility of early retirement), whether participants had to meet other responsibilities (i.e. caring for grandchildren or other family members). In addition, some aspects should be looked at more differentially. So for instance, instead of asking for parenthood, it may be important to know, whether children still live at home or whether caring responsibilities are shared with another person. In addition, it may be important to know about the job situation of the spouse (if present). Future research should aim to incorporate these variables, as they may play a crucial role in shaping both burnout experiences and retirement planning among physicians. Moreover, intention towards early retirement was measured using a single item. This may have biased the results due to lack of variance. Future studies could include a more complex, multidimensional construct in order to investigate attitudes towards retirement. However, this is the first study, investigating the association between physician burnout and retirement planning by taking into account measurements at two time points, and with a focus on specific burnout domains in a broad sample of physicians. Further strengths of the underlying longitudinal analyses included time-varying covariates and robustness over time allowing to investigate the association of burnout and intention to retire among a broad sample of physicians over a period of four years.

### Conclusion and implications

This study highlights burnout—especially work-related burnout—as a significant factor associated with early retirement intentions among physicians. In the light of physician shortage within health care settings, and in addition to the increasing numbers of physicians that will reach usual retirement age within the next years, it is important to investigate factors that may be associated with the decision to retire—especially the intention to retire early. Hence, the issue of physician retirement and burnout remains important for patients as well as health care providers.

In order to avoid negative consequences for the broader healthcare system, it is important to establish interventions that help physicians to cope with high workload. Tailored intervention should focus on improving working conditions, as well as possibilities for detachment and recovery from work [69]. In this context, changes to work structures and leadership practices can reduce burnout and the desire to leave clinical work. According to the current findings, interventions may specifically focus on work-related burnout—due to the association with early retirement. It may be important to offer both—preventive interventions (reducing the risk of burnout before it manifests) and curative interventions (addressing existing burnout symptoms).

While person-centered interventions such as resilience training or mindfulness may offer benefits.

[51, 70–72], they should complement—rather than replace—systemic solutions. Preventive efforts should also begin early, for example during medical training, to build awareness of burnout risks and healthy coping strategies from the start. Responsibility for addressing physician burnout should not lie with individuals alone. On an organizational level, it has been shown that workflow interventions may help to improve physician burnout and reduce intention to leave [73]. Employers, supervisors, and professional associations must take active roles in creating supportive, sustainable work environments. This includes advancing leadership quality, offering structured peer support, and promoting flexible work arrangements, especially for late-career physicians. For self-employed physicians, professional bodies can provide guidance and support networks to buffer against burnout[74–76]. At the same time, burnout risks and prevention strategies should be addressed long before – for example in medical schools – to make physicians aware of negative consequences of workload and possible coping mechanisms [23, 77].

To mitigate the risk of early retirement due to burnout, coordinated efforts at multiple levels of the healthcare system are required. Future studies should further explore how structural, organizational, and individual factors interact to shape burnout trajectories and retirement behavior among physicians.

## Supplementary Information

Supplementary Material 1.

## Data Availability

The data that support the findings of this study are available from the corresponding author, [F.U.J.], upon reasonable request due to ethical and privacy restrictions.
