# Supplementary material for: Exploring the link between physician burnout and intentions to retire early
Source: BMC Public Health. 2025 Oct 21;25:3557. doi: 10.1186/s12889-025-24841-3 (PMC12542239; doi:10.1186/s12889-025-24841-3)
Supplement: Supplementary file 1 — Supplementary Material 1. [file 12889_2025_24841_MOESM1_ESM.docx]

Appendix

Copenhagen Burnout Inventory (adapted for the work with patients)

Personal Burnout

1. How often do you feel tired?
2. Howoften do you feel physicalls exhausted?
3. How often do you feel mentally exhausted?
4. How often do you think: „I can’t take it anymore“?
5. How often do you feel worn out?
6. How often do you feel weak and susceptible to illness?

Work-related Burnout

1. Is your work emotionally exhausting?
2. Do you feel burnt out because of your work?
3. Does your work frustrate you?
4. Do you feel worn out at the end of the working day?
5. Are you exhausted in the morning at the thought of another day at work?
6. Do you feel that every working hour is tiring you?
7. Do you have enough energy for family and friends during leisure time?

Patient-related Burnout

1. Do you find it hard to work with patients?
2. Do you find it frustrating to work with patients?
3. Does it drain your energy to work with patients?
4. Do you feel that you give more than you get back when you work with patients?
5. Are you tired of working with patients?
6. Do you sometimes wonder how long you will be able to continue working with patients?
